# COVID-19 prevention training with video-based feedback in nursing homes: impact on staff safety behaviors

**DOI:** 10.1017/ice.2025.77

**Published:** 2025-07

**Authors:** Victoria Ngai, Joshua B. Hsi, Raveena D. Singh, John E. Mitchell, Raheeb Saavedra, Shruti K. Gohil, Emily A. Hsi, Robert Pedroza, Chase Berman, Kristine P Nguyen, Matthew Zahn, Emily Fonda, Susan S. Huang, Gabrielle M. Gussin

**Affiliations:** 1 Division of Infectious Diseases, University of California Irvine School of Medicine, Irvine, CA, USA; 2 Columbia University in the City of New York, New York, NY, USA; 3 Orange County Health Care Agency, Santa Ana, CA, USA; 4 CenCal Health, Santa Barbara, CA, USA

## Abstract

**Objective:**

Evaluate impact of COVID-19 prevention training with video-based feedback on nursing home (NH) staff safety behaviors.

**Design:**

Public health intervention

**Setting & Participants:**

Twelve NHs in Orange County, California, 6/2020-4/2022

**Methods:**

NHs received direct-to-staff COVID-19 prevention training and weekly feedback reports with video montages about hand hygiene, mask-wearing, and mask/face-touching. One-hour periods of recorded streaming video from common areas (breakroom, hallway, nursing station, entryway) were sampled randomly across days of the week and nursing shifts for safe behavior. Multivariable models assessed the intervention impact.

**Results:**

Video auditing encompassed 182,803 staff opportunities for safe behavior. Hand hygiene errors improved from first (67.0%) to last (35.7%) months of the intervention, decreasing 7.6% per month (OR = 0.92, 95% CI = 0.92–0.93, *P* < 0.001); masking errors improved from first (10.3 %) to last (6.6%) months of the intervention, decreasing 2.3% per month (OR = 0.98, 95% CI = 0.97–0.99, *P* < 0.001); face/mask touching improved from first (30.0%) to last (10.6%) months of the intervention, decreasing 2.5% per month (OR = 0.98, 95% CI = 0.97–0.98, *P* < 0.001). Hand hygiene errors were most common in entryways and on weekends, with similar rates across shifts. Masking errors and face/mask touching errors were most common in breakrooms, with the latter occurring most commonly during the day (7A.M.–3P.M.) shift, with similar rates across weekdays/weekends. Error reductions were seen across camera locations, days of the week, and nursing shifts, suggesting a widespread benefit within participating NHs.

**Conclusion:**

Direct-to-staff training with video-based feedback was temporally associated with improved hand hygiene, masking, and face/mask-touching behaviors among NH staff during the COVID-19 pandemic.

## Introduction

The COVID-19 pandemic heavily impacted NHs and underscored the necessity of proper infection prevention and control measures in this high-risk setting.^1,2^ In the U.S., NHs faced disproportionately high rates of COVID-19, exacerbated by a high-turnover workforce and limited infection prevention and control infrastructure.^3–6^

During the pandemic, resident and staff support was affected by cessation of volunteer, trainee, and visitor access.^7,8^ Pre-existing staff shortages were made worse by COVID-19 illness, which necessitated furlough for at least 10 days.^9–12^ While COVID-19 prevention was a top priority, public health personnel were diverted to outbreak response with fewer opportunities to support prevention activities.^13–15^ On-site in-person training became nearly impossible to obtain, especially for staff on night and weekend shifts.

In May of 2020, the Nursing Home COVID-19 Prevention Team was established for Orange County, CA, on behalf of the Orange County Health Care Agency and CalOptima (Orange County Medicaid). Webinars, consultative sessions, a COVID-19 helpline for NH staff, and an online toolkit (https://www.ucihealth.org/stopcovid) with training videos and protocols were provided to all 73 nursing homes in the County.^15–19^ In addition, an enhanced COVID prevention and staff safety training program was provided to 12 NHs on a volunteer basis. Here, we report the impact of the enhanced training and support program on staff behavior during the COVID-19 pandemic.

## Methods

### Staff training

The enhanced COVID-19 prevention and staff safety training program invited NHs based on their initial volume of COVID-19 cases and number of Medicaid-insured residents until 12 participants were obtained. The program involved a rolling launch from June 2020 to September 2020. Each NH received three in-person direct-to-staff training sessions (all shifts) focused on (1) staff safety, (2) proper use of personal protective equipment, and (3) environmental cleaning (relevant work shifts). A major emphasis was placed on breakroom safety due to the congregating of staff combined with mask removal to eat or drink. All NHs received posters to display in staff common areas encouraging hand hygiene, proper mask-wearing, and refraining from face/mask-touching. A breakroom safety poster reminded staff to sit 6 feet apart, to clean the table area before eating, to practice hand hygiene before removing their face mask, and to store masks in clean bags while eating, rather than placing masks on breakroom tables. Posters are included in the Supplemental Materials.

This work was conducted as a non-research public health endeavor and approved by each NH’s Quality Assurance Committee.

### Video-based feedback

We collected observations on staff safety behaviors through video-based auditing. Staff at participating NHs were informed about the activity prior to camera installation. At each NH, Google Nest (Google, California) cameras were installed in 3 locations: the staff breakroom and two non-breakroom locations (hallway, entryway, or nursing station). No cameras were installed in resident care areas.

**Figure 1.**
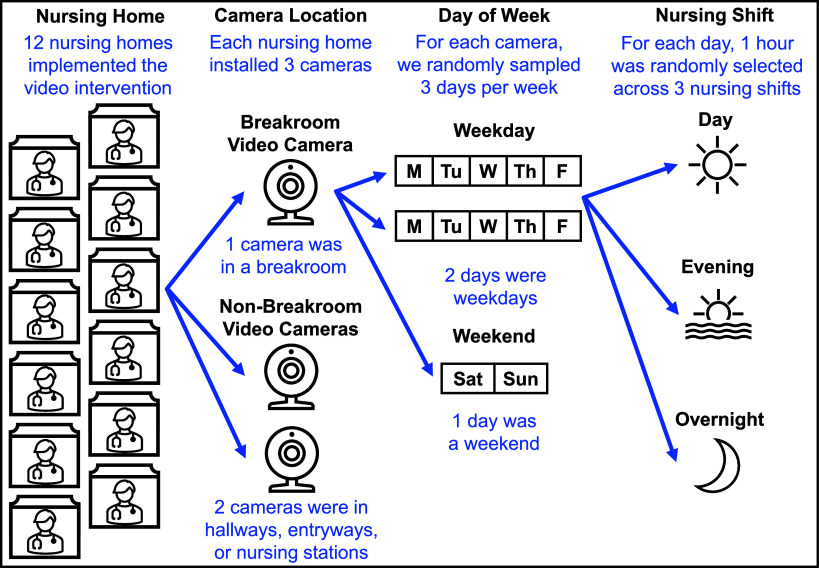
Randomization scheme for weekly 1-hour samples of video footage. Depiction of camera locations and randomized sampling scheme for review of recorded streaming video. For each camera, 1-hour blocks of footage were sampled per week with a 2:1 ratio of weekdays to weekends, evenly distributed across 3 nursing shifts: day (7A.M.–3P.M.), evening (3P.M.–11P.M.), and overnight (11P.M.–7A.M.).

NH footage (Figure 1) was audited by trained video reviewers on a weekly basis from June 2020 to April 2022. Each week, video reviewers audited nine 1-hour blocks of stored footage per camera, evenly distributed across 3 work shifts (day: 7A.M.–3P.M., evening: 3P.M.–11P.M., and overnight: 11P.M.–7A.M.), with a 2:1 ratio of weekdays to weekends. Prior to randomization, time blocks with consistently sparse video activity (eg, shifts where staff were rarely present in camera locations) were eliminated.

Determinations of correct vs incorrect staff safety behaviors were recorded in a standardized REDCap database.^20^ For each block of footage, video reviewers recorded the total number of staff observations until 25 observations were reached or 60 min elapsed, whichever came first. An observation was defined as a moment when a staff member entered into view, regardless of whether they had previously entered into view. Video reviewers tallied eligible observations of staff safety behaviors within 3 domains: hand hygiene, mask-wearing, and face/mask-touching, and determined if behaviors were correct or incorrect. Six metrics of adherence were used to measure these behaviors and evaluate performance improvement in response to training and feedback.

Hand hygiene metrics were stratified by alcohol hand rub and hand washing opportunities. The former measured the proportion of total attempts in which staff cleaned all surfaces of their hands. The latter measured the proportion of total attempts in which staff applied soap to all hand surfaces and washed for 20 seconds or longer.

Due to universal masking requirements during the COVID-19 pandemic, metrics for mask-wearing involved all observed staff except those eating and drinking in the breakroom. Proper mask-wearing required coverage of nose and mouth. The frequency of face/mask-touching without performing hand hygiene immediately before and after was assessed among all observed staff.

NHs received weekly safety report cards about staff mask-wearing, hand hygiene, masking, face/mask touching, and breakroom behavior including social distancing while unmasked assessed via video-based auditing of staff safety practices in breakrooms and common areas. In addition, video reviewers created NH-specific video montages to highlight correct and incorrect examples of staff behavior from that week. Video montages included green or red circles/arrows with corresponding subtitles to promote correction of staff safety errors and to celebrate safe behaviors. NH leadership at each facility reviewed weekly feedback and shared video montages during staff huddles.

### Analysis

We evaluated the impact of COVID-19 prevention training with video-based feedback on outcomes of (1) hand hygiene errors, (2) mask-wearing errors, and (3) face/mask-touching errors among staff in participating NHs. Video audit data were cleaned prior to analysis to remove observations with missing or illogical data.

For each staff safety metric, descriptive statistics were generated overall and for individual NHs, and graphed over time by camera location, day of week, and nursing shift.

Generalized linear mixed models were generated to evaluate variables associated with staff safety behaviors. Separate models were generated for hand hygiene, mask-wearing, and face/mask-touching. Hand hygiene models evaluated episodes of improper hand sanitizing or handwashing. Mask-wearing models evaluated episodes in which staff did not wear a mask or staff wore a mask that did not fully cover nose and mouth areas. Face/mask-touching models evaluated episodes in which staff touched their face or mask without performing hand hygiene immediately prior. Models accounted for clustering by NH and used the logit link function to predict binary outcomes. Mask-wearing and face/mask-touching models analyzed data from June 2020 to April 2022. The hand hygiene model analyzed data starting in October 2020-April 2022, when systematic hand hygiene observations began.

Covariates considered in each model included time in months from the start of the intervention (relative for each NH), camera location (hallway (referent), breakroom, entryway, nursing station), day of week (weekday (referent) or weekend), and nursing shift (day 7A.M.–3P.M. (referent), evening 11P.M.–7A.M., overnight 11P.M.–7A.M.), and busyness (entries per hour). Covariates were retained in multivariable models unless there was evidence of collinearity. Results were reported as odds ratios (ORs) with 95% confidence intervals, with ORs greater than 1 indicating more staff safety errors. Statistical significance was determined using alpha = 0.05. All analyses were conducted with SAS version 9.4 (SAS Institute, Cary, North Carolina).

## Results

Twelve NHs (Table 1) were enrolled for COVID-19 prevention training with video-based feedback. Five NHs launched by June 2020, two by July 2020, four by August 2020, and one by September 2020. Training and feedback were active in all 12 NHs by October 2020.

**Table 1.** Characteristics of participating nursing homes

Characteristic	Median (Interquartile Range) Across Nursing Homes
Number of Nursing Homes	12
Licensed Beds	98.0 (83.3–110.5)
Mean Daily Census	86.0 (76.0–116.3)
Mean Age in Years	79.5 (73.5–81.4)
% Female	55.6% (52.1–61.7%)
% Medicaid	56.8% (25.0–79.4%)
% Diabetes	40.1% (33.8–48.2%)
% Chronic Lung Disease	19.8% (17.2–22.8%)
% Renal Failure	20.7% (19.1–23.7%)
% Long-Stay (>100 Days)	57.5 (53.8–60.3)
Median Length of Stay in Days	207.0 (198.5–226.0)
Resource Utilization Group III Score^27^	1.4 (1.3–1.7)
Number of Staff Observations	15,1623 (12,841–16,466)
Hours of Footage Reviewed	690 (604.5–760)

Over 22 months (June 2020–April 2022), 8,881 randomized blocks of video footage were reviewed and over 800 report cards and video montages were returned to participating NHs. In general, in these highly trafficked areas, numbers of desired and undesired behaviors were sufficiently seen within a thirty-minute window, amounting to fifteen minutes of surveillance at 2x speed. After data cleaning, 8,279 blocks of footage (93.2%) were retained for analysis, including 182,803 staff entries, 4,652 handwashing attempts, and 3,366 hand sanitizing attempts. We note that two NHs did not have cameras with visible handwashing stations and in those NHs, only hand sanitizing was assessed.

**Figure 2.**
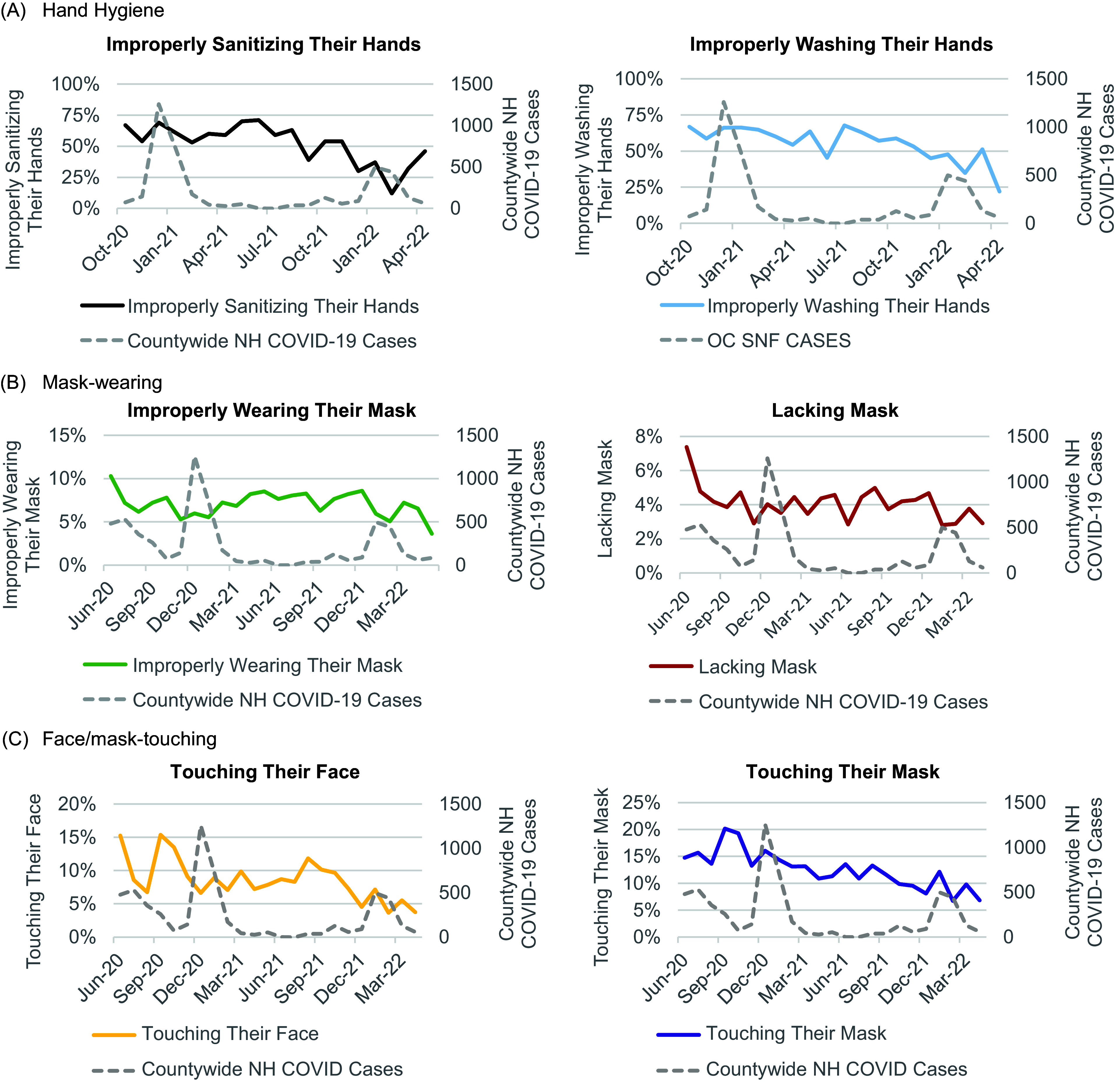
Staff safety behaviors relative to COVID-19 surges in Nursing Homes (NHs). Dual-axis line charts were used to visualize the monthly average proportions of staff safety behavior metrics (solid lines) relative to the monthly counts of countywide COVID-19 cases in NHs (dashed lines). Staff safety metrics were separated into 3 domains: (A) hand hygiene, (B) mask-wearing, (C) and face/mask-touching. (A) The average proportions of staff improperly sanitizing their hands (left) and staff improperly washing their hands (right) improve, decreasing over time (October 2020–April 2022). Hand hygiene observations began in October 2020. (B) The average proportions of staff improperly wearing their mask (left) and staff lacking masks (right) improve, decreasing over time (June 2020–April 2022). (C) The average proportions of staff touching their face (left) and staff touching their mask (right) improve, decreasing over time (June 2020–April 2022). All staff safety behavior trends do not appear to correlate with COVID-19 surges in NHs. COVID-19 case counts were retrieved from the Orange County GIS (geographic information system) open data portal.^9^

**Figure 3.**
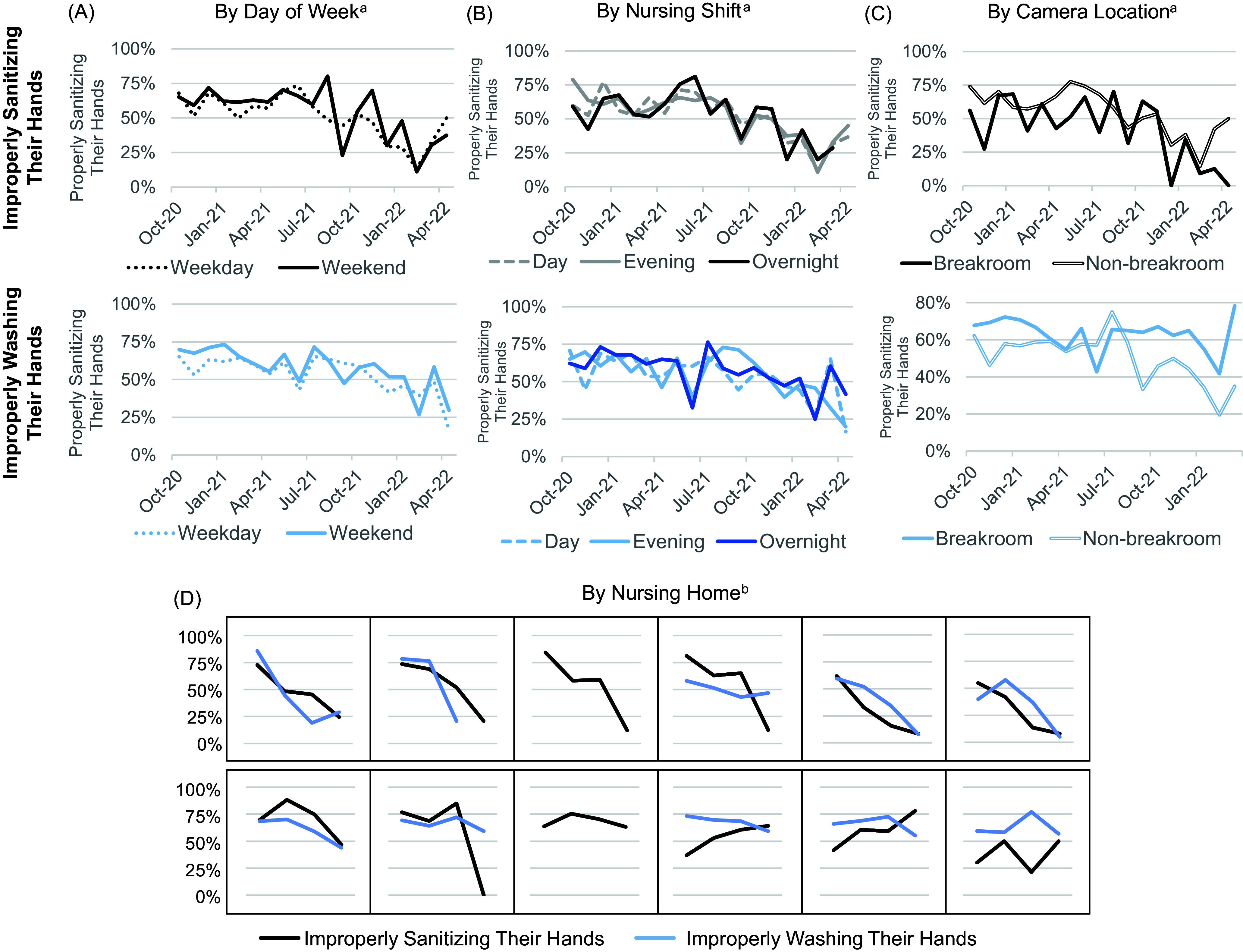
Staff hand hygiene behaviors by day of week, nursing shift, camera location, and nursing home (NH). Dual axis line charts were used to visualize the average proportions of staff hand hygiene metrics over time (October 2020–April 2022) by (A) day of week, (B) nursing shift, (C) camera location, and (D) NH. A decrease in staff errors in hand sanitizing (black lines) or handwashing (blue lines) reflected an improvement in staff safety behavior. Both hand sanitizing and handwashing improved over time on weekdays (dotted lines) and weekends (solid lines) in Panel A, as well as over time during day (dashed light lines), evening (solid light lines), and overnight shifts (solid dark lines) in Panel B. Both metrics improved over time in breakrooms (solid lines) and non-breakroom locations (hollow lines) in Panel C. In addition, metrics improved over time for most NHs as shown in Panel D. ^a^Average proportions were calculated by month. ^b^Average proportions were calculated by phases: Winter Surge (Oct 2020-Jan 2021), Rising Vaccination Rates (Feb 2021-May 2021), Delta Wave (June 2021-Nov 2021), and Omicron Wave (Dec 2021-Apr 2022). ^c^Handwashing attempts were not observed at two NHs due to camera setup. Hand hygiene observations were collected from October 2020 to April 2022.

Hand hygiene error improved over time from 67.0% in the first month of evaluation to 35.7% in the last month, decreasing independently of fluctuations in countywide NH COVID-19 cases (Figure 2). These improvements were consistent across breakroom and non-breakroom camera locations, days of the week, nursing shifts, and most participating NHs (Figure 3). In multivariable models clustered by NH and accounting for camera location, day of week, and nursing shift (Table 2), the likelihood of hand hygiene errors decreased by 8% per month (OR = 0.92, 95% CI = 0.92–0.93, *P* < 0.001). Compared to hallways, staff were 33% more likely to err in hand hygiene in entryways (OR=1.33 (95% CI = 1.11–1.58), overall *P* < 0.001), a finding that led us to restructure staff check-in areas and place hand sanitizer in front of mask boxes with signage to perform hand hygiene before taking a clean mask. Staff were 15% more likely to err in hand hygiene on weekends vs weekdays (OR = 1.15 (95% CI = 1.05–1.27, *P* = 0.004), with no significant differences across nursing shifts (Table 2).

**Table 2.** Multivariable regression for factors associated with staff safety errors

Model Outcomes and Evaluated Variables^a^	Odds Ratio (95% CI)	P-value
**Hand Hygiene Errors**		
Time in Months from Intervention Start	0.92 (0.92–0.93)^b^	P < 0.001
Camera Location		
Hallway (Referent)	1	P < 0.001
Breakroom	0.91 (0.78–1.06)	
Entryway	1.33 (1.11–1.58)	
Nursing Station	0.94 (0.77–1.14)	
Day of Week		
Weekday (Referent)	1	P = 0.004
Weekend	1.15 (1.05–1.27)	
Nursing Shift		
Day (7A.M.–3P.M.) (Referent)	1	P = 0.53
Evening (3P.M.–11P.M.)	1.06 (0.96–1.18)	
Overnight (11P.M.–7A.M.)	1.04 (0.92–1.18)	
**Masking Errors** ^c^		
Time in Months from Intervention Start	0.98 (0.97–0.99)^d^	P < 0.001
Camera Location		
Hallway (Referent)	1	P < 0.001
Breakroom	7.55 (6.70–8.50)	
Entryway	2.45 (2.13–2.81)	
Nursing Station	1.25 (1.05–1.48)	
Day of Week		
Weekday (Referent)	1	P = 0.47
Weekend	1.02 (0.97–1.09)	
Nursing Shift		
Day (7A.M.–3P.M.) (Referent)	1	P = 0.15
Evening (3P.M.–11P.M.)	0.97 (0.90–1.04)	
Overnight (11P.M.–7A.M.)	1.05 (0.97–1.14)	
**Face/Mask-Touching Errors**		
Time in Months from Intervention Start	0.98 (0.97–0.98)^e^	P < 0.001
Camera Location		
Hallway (Referent)	1	P < 0.001
Breakroom	2.93 (2.84–3.03)	
Entryway	1.00 (0.95–1.04)	
Nursing Station	1.38 (1.32–1.44)	
Day of Week		
Weekday (Referent)	1	P = 0.13
Weekend	0.98 (0.96–1.01)	
Nursing Shift		
Day (7A.M.–3P.M.) (Referent)	1	P < 0.001
Evening (3P.M.–11P.M.)	0.93 (0.90–0.95)	
Overnight (11P.M.–7A.M.)	0.89 (0.86–0.91)	

a Busyness (entries per hour) was collinear with nursing shift; nursing shift was retained in the model.

b Interpreted as an 8% reduction per month in hand hygiene errors since intervention start.

c Includes failing to wear a mask or failing to wear it properly over both nose and mouth.

d Interpreted as a 2% reduction per month in masking errors since intervention start.

e Interpreted as a 2% reduction per month in face/mask touching errors since intervention start.

**Figure 4.**
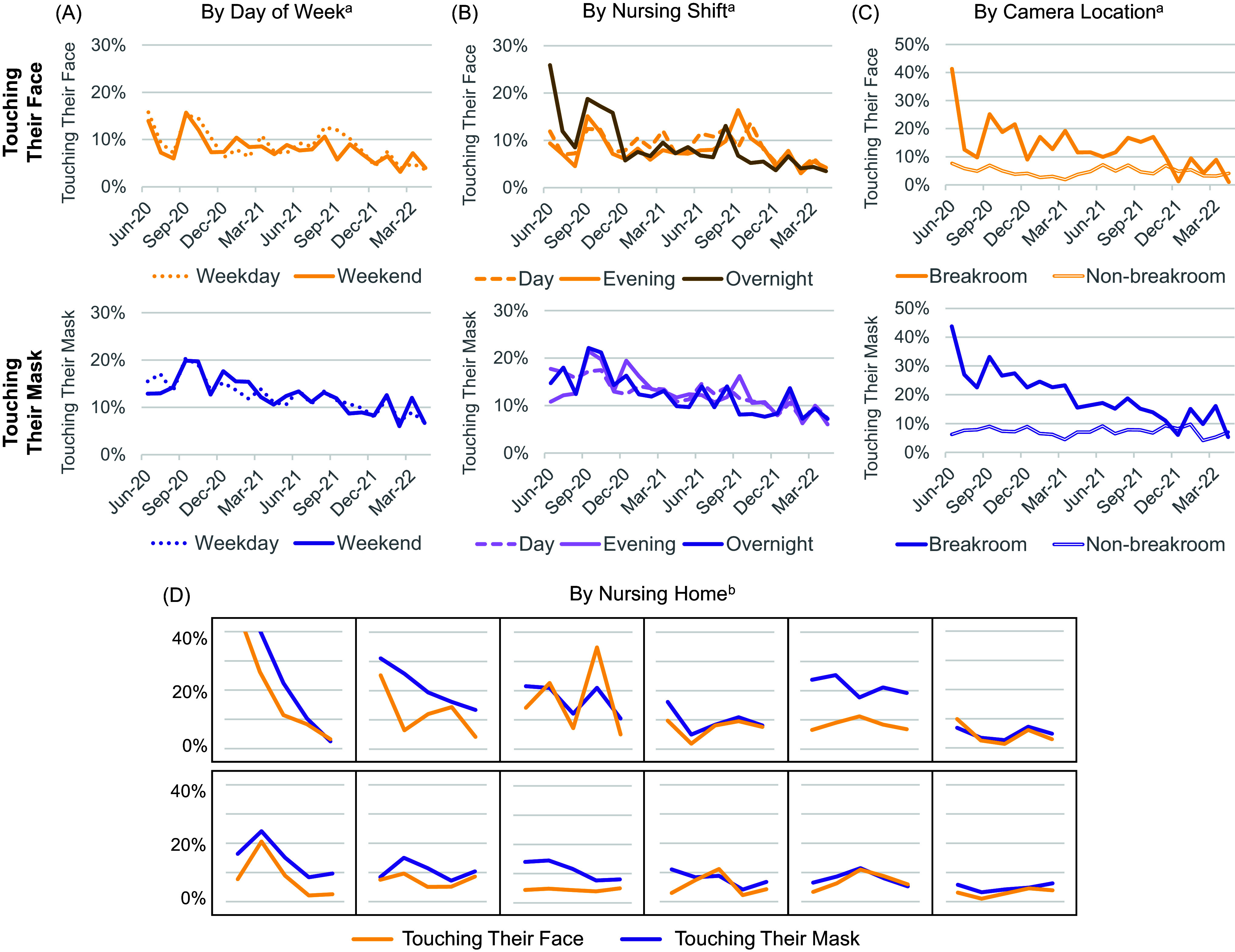
Staff face/mask-touching behaviors by day of week, nursing shift, camera location, and nursing home. Dual-axis line charts were used to visualize the average proportions of staff face/mask-touching metrics over time (June 2020–April 2022) by (A) day of week, (B) nursing shift, (C) camera location, and (D) nursing home. A decrease in staff touching their face (yellow lines) or mask (purple lines) reflected an improvement in staff safety behavior. Both face and mask touching improved over time on weekdays (dotted lines) and weekends (solid lines) in Panel A. Both metrics improved over time during day (dashed light lines), evening (solid light lines), and overnight shifts (solid dark lines) in Panel B. In addition, both metrics improved over time in breakrooms (solid lines) but were relatively unchanged in non-breakroom locations (hollow lines). In Panel D, each graph displays both metrics over time for one NH, with most showing improvement. ^a^Average proportions were calculated by month. ^b^Average proportions were calculated by phases: Program Rolling Launch (June 2020–Sep 2020), Winter Surge (Oct 2020–Jan 2021), Rising Vaccination Rates (Feb 2021–May 2021), Delta Wave (June 2021–Nov 2021), and Omicron Wave (Dec 2021–Apr 2022).

Mask-wearing errors exhibited a rapid short-term improvement from 10.3% in June 2020 to 7.2% in September 2020, after which mask-wearing errors remained low until the end of the intervention (<9%) (Figure 4). These improvements continued through waves of countywide NH COVID-19 cases (Figure 2). In multivariable models (Table 2), the likelihood of mask-wearing errors decreased by 2% per month (OR = 0.98, 95% CI = 0.97–0.99, *P* < 0.001). Compared to hallways, staff were much more likely to err in mask-wearing in breakrooms (OR = 7.55 (95% CI = 6.70–8.50), entryways (OR = 2.45 (95% CI = 2.13–2.81)), and nursing stations (OR = 1.25 (95% CI = 1.05–1.48)). Masking errors were equally likely on weekends vs weekdays and across nursing shifts.

Face/mask touching decreased over time from 30.0% in the first month of evaluation to 10.6% in the last month, improving despite fluctuations in countywide NH COVID-19 cases (Figure 2). Improvements were most evident in breakroom locations and were consistent across days of the week, nursing shifts, and most participating NHs (Figure 4). In multivariable models (Table 2), the likelihood of face/mask-touching decreased by 2% per month (OR = 0.98, 95% CI = 0.97–0.98, *P* < 0.001). Compared to hallways, staff were more likely to touch their face/mask in breakrooms (OR = 2.93 (95% CI = 2.84–3.03) and nursing stations (OR = 1.38 (95% CI = 1.32–1.44)). Face/mask-touching was less common during evening (OR = 0.93 (95% CI = 0.90–0.95) and overnight shifts (OR = 0.89 (95% CI = 0.86–0.91) compared to day shifts.

### Additional support to enhance staff safety

In response to commonly observed unsafe behaviors, we identified areas where hand-sanitizing dispensers were lacking and worked with NHs to install additional dispensers. Second, we encouraged sites to limit the number of chairs in breakrooms and position chairs to promote social distancing. Third, we worked with NHs to ensure clean paper bags were available in breakrooms for staff to safely store their masks while eating or drinking. Fourth, we worked with NHs to make disinfectant spray or wipes available in breakrooms for staff to sanitize tables before and after use. Fifth, we re-organized the check-in station in entryways to place hand sanitizer in front of mask boxes to encourage staff to perform hand hygiene before taking a clean mask. In addition, we provided “COVID-19 Hero” lapel pins for NH leaders to name and celebrate a “Staff Safety Champion of the Week.”

## Discussion

The COVID-19 pandemic underscored the critical need to bolster infection prevention and control processes in NHs. Prevention of COVID-19 and other contagious threats requires vigilant adherence to hand hygiene, use of personal protective equipment, and prevention activities that rely heavily on proper behavior. For this reason, successful implementation of these strategies requires ongoing monitoring and feedback, especially given high levels of turnover among NH staff.^21–23^ We found that real-time video-based auditing and weekly feedback reports with video montages of unsafe staff behaviors enabled improvement across the pandemic, raising adherence to key infection prevention practices.

Importantly, improvement was seen in all measured staff behaviors, with every percentage point of increased safe behavior being critical to limiting the spread of a lethal respiratory virus, especially when therapeutics and vaccines were unavailable early in the pandemic. In fact, we previously showed that this enhanced COVID-19 prevention training program successfully decreased COVID-19 cases among residents and staff.^24^

Video surveillance identified critical locations for improvement. Errors in both hand hygiene and mask-wearing were commonly seen at entryway stations where masks were donned. This likely reflected the need to establish new behavior under universal masking requirements and prompted the placement of point-of-use signage and readily available hand sanitizer. Mask errors were also particularly common in breakrooms where unmasking was expected, but attention to safe handling and storage of masks was not ingrained. Anticipated distractions related to eating, drinking, and socializing warranted additional signage and training to improve staff safety in this location. Conversely, hand hygiene and masking errors were least common in hallways, possibly because of greater visibility to other staff. Safety errors also varied by day of the week and nursing shift. Hand hygiene errors were more common on weekends, a time when NH leaders and supervisors are often not present. Face/mask touching errors were most common during day shifts, which is the busiest time for hands-on caregiving and may reflect increased work-related activity and stress among frontline staff. The day shift is also when temperatures are relatively higher and may contribute to more frequent face touching to wipe away sweat or reposition slipping masks. Nonetheless, improvements in staff safety behaviors were observed across all NH areas, staff shifts, and days of the week, supporting the widespread benefit of 24/7 random monitoring and feedback using video clip examples from all areas and shifts.

The use of video surveillance has several advantages. First, it provided an infrastructure for infection prevention support when staffing was limited given pandemic restrictions on visitors and volunteers. Second, it allowed surveillance and monitoring to be provided for all shifts across all days of the week, by allowing random sampling and review of stored video to provide a comprehensive NH intervention. This afforded a situation rarely attained since most monitoring in healthcare generally occurs in-person during weekday daytime shifts. Third, it may have prevented bias that can occur during in-person monitoring, specifically the Hawthorne effect whereby the presence of the observer improves staff behavior.^25^ Fourth, it enabled us to create quantifiable metrics for evaluating performance and improvement of staff safety behaviors using the denominator of staff opportunities/entries into the video. Fifth, while this effort focused on hand hygiene, mask-wearing, and avoidance of face/mask-touching to prevent COVID-19, the video-based approach presented here can be applied by NH leadership for pandemic or non-pandemic interventions. Video surveillance with stored footage allows flexible and customizable review whereby an intervention could be monitored more frequently during launch and less frequently during maintenance to provide counts of observed breaches or screenshots of desired and undesired behavior.

These findings also have important limitations. First, while staff were informed of the intervention and allowed us to record streaming video in healthcare provider common areas, this was likely made more acceptable due to pandemic circumstances. Thus, applicability and acceptance under normal operations remains to be seen. Second, the study design did not include a control group nor a baseline period for comparison. It is possible that the improvements seen were simply related to secular trends whereby all healthcare providers became more tolerant of mask-wearing and more conversant with proper donning procedures. However, we know that behavior change is fraught with recidivism and infection prevention lapses and outbreaks were persistently reported in NHs throughout the pandemic.^2,26^ The steady improvement in behavior despite fluctuating waves of COVID-19 combined with previously published findings that this enhanced COVID-19 prevention program reduced resident and staff COVID-19 cases compared to nonparticipating NHs in the same county help support that some of these changes in prevention behavior were induced by the program.^24^

In conclusion, a public health intervention in 12 NHs successfully used video-based auditing to assess over 8,000 hours of footage and over 180,000 staff observations to provide feedback reports and video montages of safe and unsafe behavior during the COVID-19 pandemic. This intervention was temporally associated with improvements in staff hand hygiene, proper masking, and reduced mask/face touching throughout the intervention and may provide a method for remote monitoring across multiple locations, staff shifts, and days of the week. Differential behavioral lapses by location, time of day, and day of week suggest that comprehensive infection prevention surveillance is needed during both pandemic and non-pandemic settings.

## Supporting information

Ngai et al. supplementary material 1 Ngai et al. supplementary material

Ngai et al. supplementary material 2 Ngai et al. supplementary material

## Supplementary material

The supplementary material for this article can be found at https://doi.org/10.1017/ice.2025.77
